# A Virtual Case Presentation Platform: Protocol Study

**DOI:** 10.3390/mps7020023

**Published:** 2024-03-08

**Authors:** Imad Alex Awada, Adina Magda Florea, Alexandru Scafa-Udriște

**Affiliations:** 1 Faculty of Automatic Control and Computers, National University of Science and Technology POLITEHNICA Bucharest, 060042 Bucharest, Romania; alex.awada@upb.ro (I.A.A.); adina.florea@upb.ro (A.M.F.); 2 Faculty of Medicine, University of Medicine and Pharmacy “Carol Davila” Bucharest, 050474 Bucharest, Romania

**Keywords:** e-learning, education in medical science, simulation engine, human-computer interaction, web interfaces

## Abstract

Gaining practical experience is indispensable for medical students. Therefore, when medical students were prevented access to hospitals during the COVID-19 pandemic in Romania, there was an urgent need to find a solution that would allow medical students to develop the skills they would usually develop in hospitals but without the need to be physically present in a hospital. This was the reason behind the idea of developing a Virtual Case Presentation Platform. The platform offers the possibility for medical students to reproduce virtually, in clinically valid scenarios, the diagnostic process and treatment recommendation, as well as the interactions with patients that usually take place in hospitals using natural language through speech and text. On the platform, the students receive valuable feedback from the professors about their performance. In order to reproduce the whole targeted experience for students, without missing anything, before starting the development of the platform, it was mandatory to identify and understand all the aspects that should be covered by the platform. The proposed platform covers the different aspects that have been identified for the diagnostic process and treatment recommendation. It enables medical students to develop essential skills for their future careers as doctors.

## 1. Introduction

According to Sir William Osler, “The art of the practice of medicine is to be learned only by experience, ‘tis not an inheritance; it cannot be revealed. Learn to see, learn to hear, learn to feel, learn to smell and know that by practice alone can you become expert.” [1].

Therefore, medical theoretical courses are accompanied by different clinical activities. In these activities, medical students visit hospital wards in order to interact with patients and to observe how doctors are executing different medical acts. In addition, they participate in the diagnostic process of a patient, and they recommend a corresponding treatment plan under the supervision of one or more doctors.

During the COVID-19 pandemic, lockdowns prevented medical students from accessing hospitals and thereby developing their skills in the diagnostic process of patients, treatment-plan elaboration and interaction with patients. These skills are indispensable for their future career as doctors.

In response to this issue, a partnership was created between the National University of Science and Technology Politehnica Bucharest and the University of Medicine and Pharmacy “Carol Davila” Bucharest. The aim of this partnership is to develop the Virtual Case Platform: A Virtual Case Presentation Platform that allows medical students to develop the skills mentioned earlier in a virtual environment through clinically valid real-life scenarios.

In the early stages, the platform was developed to train medical students in the field of acute and chronic heart diseases; then, it would be extended to cover other groups of diseases in later stages. A short overview of some existing e-Health simulators is provided in Section 2. Section 3 illustrates the different aspects that the platform should cover. The design of the Virtual Cases platform is described in Section 4. Section 5 illustrates the methodology of the medical data collection and anonymization. Finally, conclusions and future work are presented in Section 6.

## 2. Existing Solutions

This section makes a short overview of some similar e-Health simulators that are available as commercial solutions or research projects.

Different studies were performed in order to measure the effectiveness of virtual cases and the degree of their acceptance by students. In [2], the authors found that virtual cases were well accepted by medical students and that they were non-inferior to traditional teaching methods. The authors evidenced the potential of virtual cases to serve as an engaging learning resource; however, they recommended more studies around the effectiveness of virtual cases in medical education. In [3], the authors found that the virtual cases effectively complement current teaching. Meanwhile, in [4], the authors found that when compared to the traditional way of learning, virtual cases can be more effective in improving the skills of medical students and that they can be at least as effective in improving the knowledge of the students. In [5], the authors found that the use of virtual interactive simulators is accepted by students and that it is accurate for clinical reasoning.

Body Interact [6] is a simulator that allows medical students and health professionals to enhance their decision-making and critical reasoning skills. It provides a library with over 10,000 scenarios in different environments: pre-hospital, emergency room and consultation. The scenarios integrate clinical cases that have been developed in collaboration with international clinical reviewers. In addition, the simulator allows educators to build their own scenarios starting from zero, as well as to edit one of the existing scenarios. It integrates more than 80 physical exam items, more than 290 complementary medical tests (imaging, lab tests, decision aids, electrophysiology and more), more than 200 actions that can be performed and more than 600 medications and prescriptions. The symptoms of patients manifest through both visual and auditory ways. In each scenario, the user can access the history of the patient, order and analyze different health investigations and assist and provide care for the patient, as well as manage long care illness. At the end of the session, the student will receive automated computer-based feedback that includes a performance score.

COVID-19 Rx: Treatment Simulations [7] is a clinical reasoning simulator that is designed to teach health professionals how to treat a patient who is infected with COVID-19. The simulator includes three virtual patients: Mary Kuri, Vivien Thomas and Bagel Mage. It simulates five pre-defined detailed cases that feature clinically valid information. The first scenario is the easiest level, while the fifth is the hardest. In the first scenario, Mary suffers from a low severity of illness. In the second scenario, Mary returns to the hospital, but this time she suffers from a medium severity of illness. In the third scenario, Vivien suffers from a high severity of illness. In the fourth scenario, due to a sudden deterioration in Vivien’s health status, he suffers from a critical severity of illness. In the fifth scenario, Bagel—an elderly patient with lost health records—is transferred to an intensive care unit in an appalling situation. The user should select what scenario he/she wants to perform. Then, the user should start with the anamnesis section in which he/she selects, from a provided list of questions, the questions that he/she wants to ask the patient. The student can then visualize the patient’s answers on the screen. The student can browse the medical records of the patient and other useful information in the patient file. In addition, the student can order, from a pre-defined list of medical investigations, the investigations that he/she would like to visualize. The user also has the option to consult a specialist regarding different topics, as well as to visualize the evolution of the patient health status by advancing the time in the simulator for a specific period of time (the simulator allows intervals that are between half an hour and six hours). At the end of the session, the student will receive automated computer-based feedback.

Web-based Simulation of Patients (Web-SP) [8] is a web-based platform that allows teachers to create, manage, use and evaluate virtual patients. The platform allows students to simulate an encounter with a patient (with access to the clinical history of the patient, physical examinations and laboratory tests, as well as treatment suggestions and more). It stimulates a higher thinking process for the students, since they have to follow their own path during the scenario, to decide what investigations they should request and to interpret all their findings. In addition, students will receive automated computer-based feedback, and they will have the opportunity to compare their performances with the performances of experts.

Compared with the Virtual Cases platform, which is still in its early stage of development, all the analyzed platforms, with the exception of Web-SP, offer a richer and more attractive interface that includes animations. However, the strong advantage of the Virtual Cases platform is the way in which the scenarios are generated in the platform. While all the reviewed platforms integrate scenarios that are based on predefined cases and do not offer the possibility to break a case down into different elements (such as history, blood tests, pulmonary auscultation and ultrasound), the Virtual Cases platform allows professors to define informative labels over each element of the case as well as to specify the restrictions that should be respected during the association of labels. Therefore, the Virtual Cases platform allows for the generation of medically pertinent cases that are not pre-defined but are based on the association of elements that are included in the platform and that come from real-life cases, based on the clinically allowed associations between their labels. Another advantage of the Virtual Cases platform is that the session evaluation report is automatically generated by combining the computer-based feedback and the professor’s feedback, while the reviewed platforms offer feedback based only on computer-based feedback. In addition, the support of the natural language interactions between the student and the virtual patient through speech and text-based interactions is another advantage of the Virtual Cases platform, particularly the interactions using the Romanian language.

## 3. Aspects That Should Be Covered by the Platform

The Virtual Cases platform should cover all the aspects of the clinical activities that occur during the process of diagnosing a patient and recommending treatment. This also includes the possibility for the doctors to evaluate the students’ performances and to provide feedback during the interaction of the students with their patients and/or after the end of the interaction.

### 3.1. Diagnostic Process and Treatment Recommendation

At the beginning of the diagnostic process, the medical student inquiries about the patient’s name, age and sex. The student observes the general appearance of the patient, which offers important clues for the diagnostic process.

The student asks the patient specific questions and receives answers in order to collect reliable/objective information about the patient and his/her health condition. If the patient is unable to take part in a conversation, the student should eventually talk to people that are close to the patient. This is known as the anamnesis stage. Mainly, but not exclusively, the questions refer to the reason for the patient’s presence in the hospital, the onset of symptoms, the evolution of symptoms, the patient’s medical history and state, their family medical history and ongoing medical treatments, as well as questions that evaluate the patient’s consciousness.

All the details are important; however, according to [9], the student should evaluate systematically the following details:

- faces and expressions: by observing the facial expression of the patient;
- gait: by observing the steps/walking process of the patient, if the patient is able to walk;
- stature and habitus of the patient: by observing the body build of the patient;
- posture and decubitus: by observing the states of the muscle groups and the posture of the patient, such as standing up, sitting down, fowler’s position, semi-fowler’s position and supine position;
- odor of breath and body: by carefully smelling the surrounding air and the breath of the patient;
- clothing and paraphernalia: by observing the clothes of the patient and any accessories.

The student should perform an objective examination, which is a practical and comprehensive assessment of the patient’s body. The objective examination varies depending on the patient’s symptoms, but mainly the student observes and notes:

- external signs of the patient such as the weight of the patient, the presence of any scar or any forms of jaundice and dyschromia on the patient’s body, as well as the presence of a deformation of the spine;
- auscultation of the respiratory system;
- auscultation of the heart (cardiac auscultation);
- other systems and organs (if needed, depending on the patient’s symptoms) such as the digestive system.

After the anamnesis stage and physical examination, the student should review the medical records of the patient and go through the various information that may be available about the patient and that may be relevant to the current state of the patient.

The student should have reached a presumptive diagnosis by now. The presumptive diagnosis is the most likely condition of the patient based on the information that the student has gathered and analyzed since the beginning of the diagnostic process.

After defining the presumptive diagnosis, the student must request various medical investigations such as electrocardiogram (EKG), radiography, computed tomography (CT), ultrasound, blood test and angiography. The requested investigations vary in relation to the presumptive diagnosis.

After receiving the requested medical investigations, the student must interpret each investigation. This is followed by a differential diagnosis that excludes alternative diagnoses with similar clinical manifestations.

In order to narrow the list of the differential diagnosis, the students may ask for additional medical investigations. After narrowing the list, the student must define the final diagnosis in which he/she can sustain the presumptive diagnosis or change it.

The next step is for the student to elaborate a treatment plan and recommend it to the patient. The treatment plan can have one or more types:

- dietetic–hygiene treatment: consists of some specific dietetic and/or hygienic measures that the patient must follow;
- pharmacological treatment: consists of some specific medications that the patient must take;
- interventional treatment: consists of minimally invasive procedures on the patient (generally alternatives to performing surgery);
- surgical treatment: consists of performing one or more surgeries on the patient.

After that, the student describes the possible complications that may appear during the illness/treatment, and he/she defines a prognosis that contains the prediction of the patient’s disease trajectory/evolution [10].

### 3.2. Possibility for Doctors to Evaluate Medical Students and to Provide Feedback

During the clinical activities of the medical students, doctors observe the interactions between the students and the patients as well as all the acts that are performed by the students during the process of diagnosing patients and recommending treatments.

The doctors can provide feedback/hints for the students during their interactions with the patients or after its end.

In addition, doctors evaluate each interaction of the students as well as every aspect of any medical act that is performed by the students.

## 4. Platform Design

This section illustrates the design of the platform.

The “Digital education for building health workforce capacity” report [11] published by the World Health Organization (WHO) in April 2020 highlights that digital education has the potential to improve the skills and satisfaction of health professionals. In addition, the report identifies that the effectiveness of digital health education varies widely depending on how it is implemented.

Multiple scientific papers [12,13,14] argue that the effectiveness and outcomes of digital health education vary widely depending on the following:

- learning modality (e.g., computers, mobile phones, tablets, gamification, serious games, virtual reality, type of interactions);
- delivery mode (e.g., fully digital, face-to-face or hybrid);
- instructional method (e.g., simulations);
- assessment methods (e.g., use of validation tools or not);
- learning pedagogies (e.g., problem-based or team-based);
- learning topics and disciplines that are being taught (e.g., smoking cessation, diabetes management, domestic violence, antibiotic management, elderly care, child health);
- access to digital education and the targeted professionals (users of digital health education).

**Figure 1:**
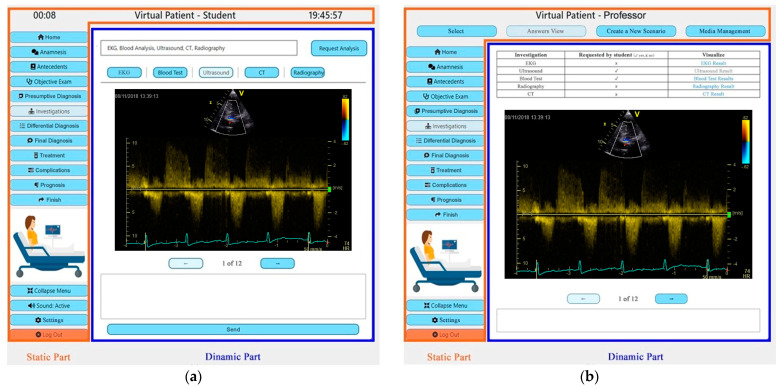
GUI, dynamic part and static part (**a**) Student View; (**b**) Professor View.

For these reasons, it was determined that the Virtual Cases platform should integrate a graphical interface (GUI) that is intuitive and easy for users. The GUI should provide quick access to all functionalities of the platform, should integrate some customizable features and should adapt itself to the screen of the device from which it is accessed as well as to the type of user. It consists of a static and a dynamic part throughout the session, as illustrated in Figure 1.

In the student view, the static part contains the clock at the top right of the screen and the current session timer at the top left of the screen. The navigation menu is found on the left side of the screen. It contains various sections that enable medical students to carry out the complete diagnostic process and treatment recommendation. Each section was created according to the process stages and goals that were identified in Section 3.1. Below the menu, there is a picture that illustrates the current position of the patient followed by a secondary menu that contains the menus mode, sound control (on/off), settings and log-out buttons, whereas the dynamic part contains the information that varies depending on the section that the student is navigating.

**Figure 2:**
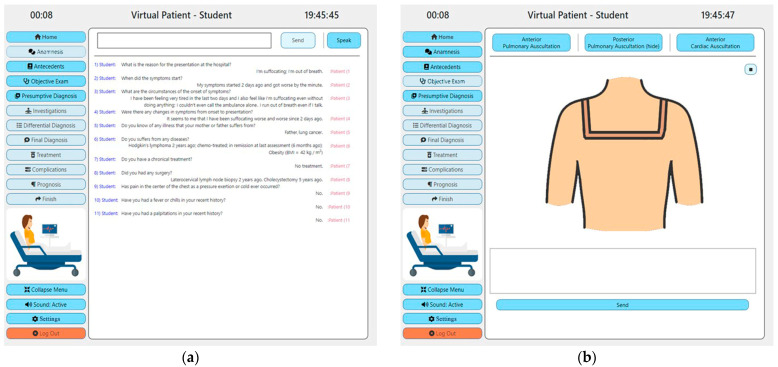
Prototype-Student View (**a**) Anamnesis Section; (**b**) Objective Exam Section.

Once the student successfully authenticates, the home section is displayed. The dynamic part of the page contains the patient’s name, age and sex as well as a picture of the patient that illustrates the current position of the patient (it allows the student to observe and evaluate the position, posture, facial expression, clothing and/or paraphernalia of the patient). The student can navigate to the anamnesis section in which he/she can ask questions to the patient using natural language through speech- or text-based interactions. Once the student finishes a question, the question will be displayed on the screen and followed by the answer of the patient as illustrated in Figure 2. The answer of the patient will be heard through the phonetic outputs of the system (unless the student has deactivated this feature). The student can navigate to the antecedents section in order to visualize the medical history of the patient. In the objective exam section that is illustrated in Figure 2, the student can perform the objective exam. For acute and chronic heart disease, the objective exam consists of anterior and posterior pulmonary auscultations and cardiac auscultation. To perform an auscultation, the student should put the mouse cursor or his finger (on touch-enabled screens) over the patient’s body in the positions in which he/she would put the stethoscope in real life, and he/she will hear through the phonetic outputs of the system the sounds that he/she would hear through the stethoscope in real life (the sound will vary in function of the chosen position and the type of auscultation). After passing through the previous sections, the student should provide a presumptive diagnosis. Once it is provided, the student cannot change it.

In the investigations section, the student should request different health investigations that he/she wants to visualize (such as electrocardiogram, radiography, computed tomography, ultrasound, blood test and angiography). After the student’s request, the requested investigations will be displayed on the screen. The student has the freedom to navigate between the different investigations and to ask for additional ones. The student should provide an interpretation for each investigation that he/she has requested. In the differential diagnosis section, the student should provide a differential diagnosis in which he/she should exclude alternative diagnoses with similar clinical manifestations to the case he/she is treating. The student should provide a final diagnosis in the final diagnosis section. In this diagnosis, the student can sustain the presumptive diagnosis that he/she provided earlier or completely change it. In the treatment section, the student should elaborate a treatment plan that can have one or more types (dietetic–hygiene, pharmacological, interventional and/or surgical). The student should describe the possible complications that may appear during the evolution of the illness and/or during treatment, in the complication section. In the prognosis section, the student should define a prognosis in which he/she predicts the trajectory/evolution of the patient’s disease.

It is worth mentioning that the student has the freedom to navigate between the first five sections of the platform in any order that he/she prefers. The rest of the sections are deactivated until the student provides the presumptive diagnosis. Once the student provides the presumptive diagnosis, the rest of the sections will be activated, and the student will have the freedom to navigate between all of them. The student also has the option to skip any section with the exception of the presumptive diagnosis section (this is mandatory to be validated in order to enable the rest of the sections). Skipping a section will be sanctioned, and it will affect the performance score of the section awarded by the platform. Another aspect that should be highlighted is the fact that only the information that is displayed in the home and antecedents sections are provided by default during each session; the student should explicitly ask for the rest of the information/data. The student will receive only the information/data that he/she asks for during the session.

In the professor view, the static part contains a horizontal menu at the top of the screen and a vertical menu at the left of the screen. The vertical menu is followed by a picture showing the position of the patient in the session that the teacher is visualizing, followed by a secondary menu that contains the menus mode, settings and log-out buttons, whereas the dynamic part contains the information that varies depending on the section that the professor is navigating.

According to the identified functionalities and needs of the Virtual Cases platform, it has been established that the platform should have three types of users. In addition to “student” and “professor” user types, “administrator” is the third user type. The view of the administrator is more simplistic since it integrates limited functionalities. The view allows the administrator users to visualize the users that are registered on the platform and to create new users as well as to edit or delete any user and to associate a student/group of students with a teacher. In addition, it allows the administrator users to manage (edit, add or remove) the diseases that are available on the platform as well as to manage the data that are associated with these diseases (such as health investigations, annotations of health investigations, scenarios).

In order to enable live feedback from the doctors during the student session and the possibility for doctors to provide feedback after the end of the student’s session, the platform should integrate two types of sessions:

- solo session type: in which a student performs a scenario alone. The student will receive the session evaluation report and the feedback of the professor after the session;
- group session type: which requires the presence of the professor and the students of the group during the performance of each student. Depending on the professor’s preferences, each student can receive the feedback of the professor live during the session or after it. Each student will receive the session evaluation report after the session.

It is important to highlight that the session evaluation report is generated automatically by the platform (computer-based feedback that includes a score). The performance score is awarded by the scoring algorithm that is integrated into the platform. The maximum score that can be awarded to a student per session is 100 points. The home and antecedents sections are not scored since if the student missed these sections, the missing information will be reflected in the answers that will be provided in the other sessions (wrong or incomplete answers), and as a consequence the score obtained for these answers will drop. For the rest of the sections of the diagnostic process and treatment recommendation, each section can have a maximum score of 11 points with the exception of the treatment section, which can have a maximum score of 12 points:

- anamnesis section (11 points): depending on the questions that were asked by the student in this section (asking all the essential questions {compared to a predefined golden-set of questions for each disease}, the order of the questions, etc.);
- objective exam section (11 points): depending on the manner in which the objective examination was performed (coverage of various aspects/auscultation points within the examination, the order of their coverage, etc.);
- presumptive diagnosis section (11 points): depending on the presumptive diagnosis provided by the student and its justification;
- investigations section (11 points): depending on the medical investigations that were requested by the student and the provided interpretation for each investigation (the request of all essential investigations {compared to a predefined golden-set of essential investigations for each disease}, the order in which the investigations were requested and the interpretation provided for each investigation);
- differential diagnosis section (11 points): depending on the differential diagnosis provided and its justification;
- final diagnosis section (11 points): depending on the final diagnosis provided and its justification;
- treatment section (12 points): depending on the elaborated treatment plan and its justification;
- complications section (11 points): depending on the description provided of the complications that may occur during the course of the disease and/or during treatment;
- prognosis section (11 points): depending on the prognosis offered and its justification.

The provided answers are compared with a set of accepted answers for each section that is associated with the scenario in the moment in which the scenario is generated (depending on the illness, its severity, the tags and annotations of the investigations that were associated with the scenario, etc.).

In addition, before awarding the final grade, the scoring algorithm takes into consideration the duration of the entire session, but also the time spent by the student in each section (if the time limit, which varies according to the scenario and the type of session, is exceeded, the student will be sanctioned by 1 point for every 5 min, and this criterion cannot bring extra points). It also takes into account any feedback provided by the professor during or after the session (which can bring minus or plus points, without the possibility of exceeding the total of 100 points).

The professor has the option to adjust the evaluation report including the score that was awarded by the platform.

The Virtual Cases platform supports natural language interactions between the student and the patient through speech- and text-based interactions, in both Romanian and English, which is another important feature of the platform.

The platform integrates a generation engine that generates clinically valid scenarios. The inputs of the engine are the disease type, the severity level of the disease and the constraints that are established by a professor for the disease type and the severity level, as well as any data that the professor wants to be included in the generated scenario (if any). To generate a new scenario, the engine assembles the different elements of the scenario based on these arguments by filtering out the clinically valid artifacts that are uploaded on the platform and that contain such labels.

**Figure 3:**
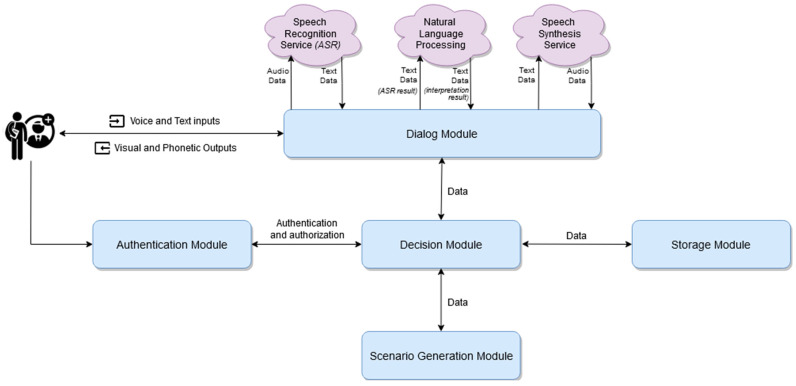
The Architecture of the Virtual Cases platform–Block Diagram.

The implementation and description of the initial version of the platform are detailed in [15]. The platform has a modular, microservice-based architecture that is illustrated in Figure 3 and is detailed in [16]. It is composed of five main modules:

- the authentication module: this is responsible for authenticating users;
- the decision module: this is the place in which the decision-making processes take place based on one or more conditions;
- the scenario generation module: this is the place that integrates the engine that generates clinically valid scenarios;
- the storage module: this is the place in which all the data is stored;
- the dialogue module: this is responsible for managing the dialogue between the student and the virtual patient as well as for storing the history of the current session’s interactions.

Another important factor that should be highlighted is that the platform uses AI techniques to draw the professor’s attention in some specific cases, such as:

- if a large number of students makes the same mistake;
- if a student makes specific mistakes repeatedly (e.g., the student always wrongly interprets a chest X-ray or if the student always misses a specific point of auscultation during the objective examination);
- if a large number of students fails to correctly complete a specific scenario (even if the reason for failure differs between students).

## 5. Medical Data Collection and Anonymization Methodology

This section describes how the medical data of the Virtual Cases platform has been and is being collected and anonymized.

Regarding the data that are associated with the anamnesis stage: for each pathology, a document has been elaborated by a team of health professionals. The document contains clinically relevant questions for anamnesis and the possible answers of the patient in relation to the severity of the illness together with the probability of each answer. The probability of the answers that are related to the symptoms, their severity and signs respect the probability of their occurrence reported in the specialized literature and in the European guidelines dedicated to each pathology [17,18,19,20].

Regarding the data that are associated with the objective examination: the data are composed of cardiac auscultation as well as anterior and posterior pulmonary auscultations. These auscultations belong to patients that were hospitalized in the Cardiology Department of the Bucharest Clinical Emergency Hospital (SCUB). The auscultations are stored in an mp3 format audio file.

Regarding the data that are associated with the medical investigations: the data are composed of various medical investigations that belong to real patients that were hospitalized in SCUB. The medical investigations are:

- electrocardiograms: scanned electrocardiograms saved as documents in pdf format or as images in jpeg format;
- transthoracic echocardiograms (ultrasounds): exported from the storage of the echocardiographs as images in jpeg, png and tif formats or as videos in mp4 format;
- chest X-rays: exported from the SCUB’s radiological investigations intranet storage, as images in jpeg, png or tif formats;
- computed tomography: exported as well from the SCUB’s radiological investigations intranet storage as images in jpeg, png or tif formats;
- blood tests: blood test results introduced in tabular format in an Excel file in xls format;
- angiographies: exported from the storage of the angiograph as videos in mp4 format;
- computed tomography angiography: exported as well from the SCUB’s radiological investigations intranet storage as videos in mp4 format.

Regarding the patient’s medical history, for each pathology, a team of health professionals collectively elaborated a document that contains various information about the medical history of patients (from the data that were available in SCUB but also from data that were extracted from the literature).

Before uploading the data, the entire data set is anonymized in order to prevent the data from being traced to their original patients: any element that can identify the patient from whom the data originate is removed.

All medical data that are uploaded to the Virtual Cases platform are clinically valid data, and they are labeled both by the disease for which they are representative and with the severity of the manifestation of that pathology as well as other useful information (e.g., pulmonary thromboembolism, high risk). Labeling each element of each medical case (including its medical data) is a necessary step in order to allow the scenario generation engine of the virtual platform to generate consistent scenarios that are clinically valid. The generation is based on the association of different elements that are included in the platform and that come from real-life cases, based on the clinically allowed associations between their labels and by respecting the restrictions that should be respected during the association of labels (and not distributed randomly). These restrictions were specified by the doctors. This ensures obtaining new cases that are similar to the real ones (clinically valid), but with certain peculiarities and degrees of difficulty.

## 6. Conclusions and Future Work

The Virtual Cases platform is able to portray real patients in clinically valid scenarios. They can engage in interactions with medical students using natural language through text and speech interactions. Medical students have the flexibility to perform the training whenever they prefer without having to be physically present in the same location as the patient and the doctor/professor. In addition, they can go through this training (including the possibility to redo the same scenario) no matter how many times they feel the need. This offers a huge opportunity for students to enhance their skills and knowledge.

The Virtual Cases platform offers the possibility for medical students to emulate interactions with patients, the diagnostic process and the recommendation of treatment that usually occur in hospitals, virtually, in clinically valid scenarios within the field of acute and chronic heart diseases. The students can receive feedback from their professors during or after the session.

As future work, the extension of the platform is envisaged in order to cover more heart diseases in nearing stages and then to cover other groups of diseases in later stages. Another envisaged task is to enable the platform to generate and support complex scenarios in which the virtual patient suffers from multiple pathologies. The use of General Adversarial Networks to generate new, synthetic realistic and consistent data and the enhancement of the platform’s GUI and the improvement of natural language interactions are also envisaged as future work.

## Data Availability

Not applicable.
